# Are common fragile sites merely structural domains or highly organized “functional” units susceptible to oncogenic stress?

**DOI:** 10.1007/s00018-014-1717-x

**Published:** 2014-09-20

**Authors:** Alexandros G. Georgakilas, Petros Tsantoulis, Athanassios Kotsinas, Ioannis Michalopoulos, Paul Townsend, Vassilis G. Gorgoulis

**Affiliations:** 1 Physics Department, School of Applied Mathematical and Physical Sciences, National Technical University of Athens (NTUA), Zografou, 15780 Athens, Greece; 2 Division of Oncology, Geneva University Hospitals, 1211 Geneva, Switzerland; 3 Molecular Carcinogenesis Group, Department of Histology and Embryology, School of Medicine, University of Athens, 11527 Athens, Greece; 4 Computational Biology and Medicine, Center of Systems Biology, Biomedical Research Foundation of the Academy of Athens, 4 Soranou Efessiou, 11527 Athens, Greece; 5 Faculty Institute for Cancer Sciences, University of Manchester, Manchester Academic Health Science Centre, Manchester, M13 9WL UK; 6 Manchester Centre for Cellular Metabolism, University of Manchester, Manchester Academic Health Science Centre, Manchester, M13 9WL UK; 7 Biomedical Research Foundation of the Academy of Athens, 4 Soranou Efessiou, 11527 Athens, Greece; 8 Department of Histology-Embryology, Medical School, National Kapodistrian University of Athens, 75 Mikras Asias Str., Goudi, 11527 Athens, Greece

**Keywords:** Fragile sites, miRNAs, Genomic instability, DNA elements, DNA repair, Carcinogenesis

## Abstract

Common fragile sites (CFSs) are regions of the genome with a predisposition to DNA double-strand breaks in response to intrinsic (oncogenic) or extrinsic replication stress. CFS breakage is a common feature in carcinogenesis from its earliest stages. Given that a number of oncogenes and tumor suppressors are located within CFSs, a question that emerges is whether fragility in these regions is only a structural “passive” incident or an event with a profound biological effect. Furthermore, there is sparse evidence that other elements, like non-coding RNAs, are positioned with them. By analyzing data from various libraries, like miRbase and ENCODE, we show a prevalence of various cancer-related genes, miRNAs, and regulatory binding sites, such as CTCF within CFSs. We propose that CFSs are not only susceptible structural domains, but highly organized “functional” entities that when targeted, severe repercussion for cell homeostasis occurs.

## Introduction

Activated oncogenes are a key feature of cancer development from its earliest stages [1]. One of their major effects is the induction of DNA damage via replication stress (RS) [2]. Specifically, oncogene-induced DNA replication stress (OIRS) leads to the formation of DNA double-strand breaks (DSBs), due to replication forks (RFs) collapse, fueling genomic instability (GI) [2, 3]. In early precancerous lesions, the collapse of DNA RFs occurs preferentially at specific loci termed fragile sites (FSs) [4]. As a result, FSs exhibit breaks, gaps, and rearrangements, collectively termed FSs expression. This is due to the activation of pathways responsible for fork collapse resolution and completion of DNA replication, which involve recombinogenic processes and DNA DSBs production [2].

**Fig. 1:**
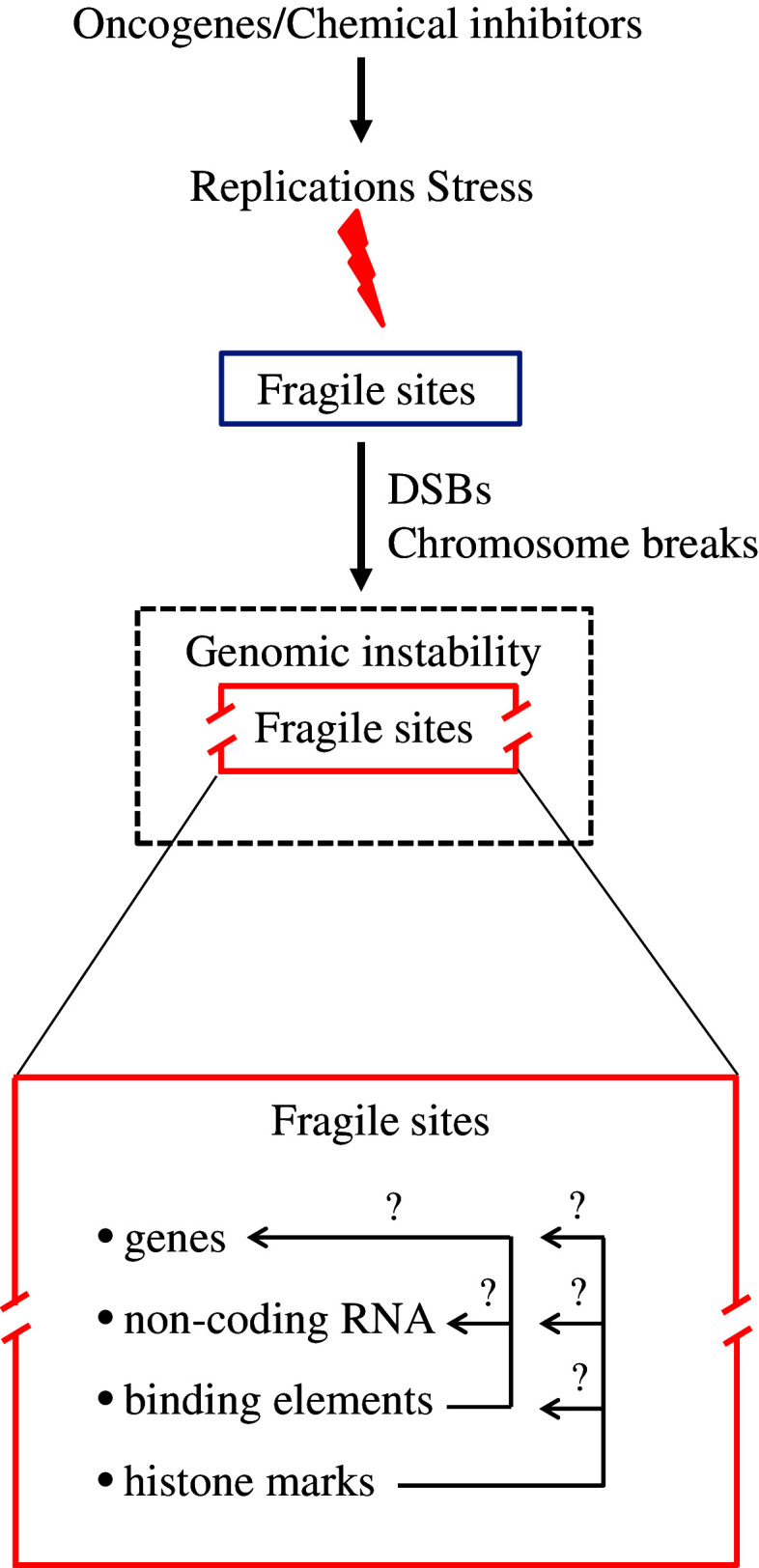
CFSs are not only vulnerable structural domains but may also be functional units of the genome that are sensitive to replication stress. CFS’s stability is affected by replications stress (RS). During cancer development, they are affected from the earliest precancerous lesions due to oncogene-induced replication stress (OIRS). Breakage at CFSs (*broken red rectangle*) may not only confer to genomic instability (GI) (*dashed black rectangle*), but could also have wider biological implications by affecting elements located within them (*question mark*). *DSBs* DNA double-strand breaks

An important issue is whether the instability manifested at these sites has any wider biological impact on cancer development. As further discussed in the manuscript, although FSs are heterogeneous in their expression patterns, they possess unique features that make them vulnerable to structural destabilization under RS conditions. Are these regions simply prone to DNA damage due to their intrinsic characteristics, conferring only to GI? Sparse evidence indicates that FSs enclose genes and non-coding RNAs, like microRNAs (miRs), while their expression could be epigenetically modulated by histones, implying that they are regions of the genome of a higher organization level (Fig. 1) [5–9]. Important bioinformatic resources are currently available and can be exploited to define potential topological associations between CFSs and these elements. Notably, the miRbase is constantly expanding while the ENCODE project [10] has deposited information on a vast range of binding elements and genomic modifications, including histone marks (like H3K79me2, H3K9ac, H3K4me3, and H3K27ac) that have a prominent influence on the expression process of the genome. As the pattern of instability at FSs in human tumors is variable, suggesting that it also depends on the cell type, this further complicates the role of FSs in malignancy. Last but not least, if such sites contribute to cancer development, why have they not been evolutionary selected for elimination? Could there be a higher reason that makes them at the same time vulnerable “units” of the genome with potentially meaningful function? Attempting to address these questions, in the current work we conducted an extensive review on the nature of their heterogeneity that accounts for their preferential instability. Next, by applying bioinformatic tools on data from the latest miRbase and the ENCODE project, we reveal that these sites are enriched in various (coding and non-coding) elements, such as cancer-related genes, miRs, and binding elements, as well as specific variations in histone modifications (Fig. 1). Based on these findings, we propose that these sites may represent unique “functional” units of the genome that may have a complex role upon OIRS with implications both in normal cell survival and cancer progression.

## Heterogeneity of fragile sites

FSs have been assigned in two classes, defined as rare fragile sites (RFSs) and common fragile sites (CFSs). Rare FSs are mainly induced by folate deficiency, correspond to dinucleotide or trinucleotide repeats, usually CGG_n_, and are found in less than 5 % of the human population and in specific families [11]. Their fragility is due to expansions of the micro- or mini-satellites sequences that they contain and in some cases are responsible for inherited diseases [11]. Therefore, RFSs will not be further discussed in this work.

Historically, CFSs were recognized as recurrent hotspots of double-stranded DNA breaks in cultured lymphocytes from healthy individuals [12]. They are present in all individuals, are part of the normal chromosomes, but exhibit different frequencies of expression in a population (reviewed in [13, 14]). CFSs are typically vulnerable to extrinsic replication stress, most notably to aphidicolin (APH), an inhibitor of DNA polymerase α, δ and ε, but are otherwise quiescent under normal conditions. This observation has been gradually broadened to include breakage patterns resulting from various replication inhibitors, such as nucleotide analogs (5-azacytidine, bromodeoxyuridine) or antitumor antibiotics (distamycin) and RS resulting from folate deficiency. Dietary and environmental factors like caffeine, cigarette smoke, and hypoxia may also enhance FS expression [15]. Recently, the induction of stress during the early S phase in B lymphocytes by hydroxyurea has been found to provoke DNA damage in a distinct pattern, corresponding to a new class of “early replicating fragile sites” (ERFSs) [16]. They occur primarily in early replicating DNA, close to replication origins, and are mainly situated in actively transcribed gene clusters (coding regions) [17]. This contrasts with CFSs, like FRA3B, which are most sensitive during their replication in late S phase [18]. Nevertheless, ERFSs seem also to arise from RF collapse and are similarly sensitive to ATR inhibition and oncogene-induced stress (see A. Nussenzweig chapter in this issue). OIRS is expected to induce instability at both ERFS and CFS, as suggested in two independent studies [16, 19]. Recent reports using the phosphorylated form of histone H2AX, the γ-H2AX, as a marker of DSB induction showed that ERFS were enriched for H2AX and γ-H2AX, while CFSs and heterochromatin lacked both, also suggesting differential DNA damage response at these sites [20]. Notably, both ERFSs and CFSs are rich in CpG-rich regions [17, 19], implying that these classes of FSs may either share structural similarities or the exact classification of their members as early and late replicating ones may need further re-assessment. Surprisingly, telomeric regions also appear to exhibit fragility in a similar manner as CFSs upon replication stress, including APH treatment [21].

It has been shown that CFS expression patterns depend not only on culture conditions but also on cell type [22]. Although traditionally studied almost exclusively in lymphocytes, different CFSs have been observed in fibroblasts [22, 23], breast, and colon epithelial cell lines [24, 25] and erythroid cell lines [25]. Considerable overlap exists between experiments, but the relative frequency of CFS breaks varies significantly. For example, FRA3B is the most frequent fragile locus in lymphocytes, but does not seem to be fragile in epithelial cell lines [24, 26], possibly due to the plasticity of replication programs in different cell lineages or because of a putative “housekeeping” role of *FHIT*. The second most fragile site, FRA16D, is very frequently affected in epithelial breast cancer cell lines (20–25 %), but only occasionally in colon epithelial cells (~5 %). Clearly, a complete characterization of CFS breakage probabilities will require a panel of different cell types.

CFSs are found in different individuals and are conserved across different species, including the mouse, the rat, and many mammals [27, 28], and across kingdoms, such as in the yeast *S. cerevisiae* [29]. Evolutionary conservation could argue in favor of a meaningful function if CFSs are considered outliers compared with the overall fragility of the genome in general. Nevertheless, variation between individuals can be significant. In a study of 20 normal adults [30], only FRA3B and FRA16D were found to be fragile in all individuals, and only 42 % of CFSs (19 of 45 identified) were present in the majority of individuals. In the earliest studies, less than 20 CFSs would explain more than 80 % of gaps and breaks [12]. A similar distribution was found in a population study of Deer mice, where high-frequency CFSs constituted approximately 26 % of the population total breaks and 38 % of CFSs were only found in single individuals.

## Fragility of CFS

The issue of fragility at CFSs is a matter of intensive investigation. CFSs replicate either late in S-phase or initiate replication in mid-S phase, but exhibit a significant delay in completing it. Under conditions of RS, they may remain unreplicated even during G2-phase and up to mitosis leading eventually to their instability (as discussed in next section and reviewed in [13, 14]). Several features responsible for their replication sensitivity have not been revealed until now. These include intrinsic structural characteristics, the presence and overlap with large genes, differences in replication features, and epigenetic modulation [13, 14].

At the structural level, CFSs have the propensity to form secondary non-B structures that interfere with the movement of the replication fork thus leading to its collapse and associated DNA breaks [31]. Specifically, at sequence level, CFS are enriched in long stretches of AT dinucleotide-rich repeats that may form stable secondary cruciform DNA structures inducing fork stalling during DNA replication and in general incomplete or delayed DNA replication [31, 32]. In an earlier study, we performed a whole-genome analysis of CFS sequences and observed that they are on average rich in GC and Alu sequences [19]. The Alu family is a family of short interspersed repetitive elements (SINE) of about 300 bp containing mid and terminal poly A-stretches [33]. Interestingly, these elements are the most abundant mobile elements, and thus potentially recombinogenic in the human genome, and are implicated in various inherited human diseases and in cancer [34].

CFSs have been associated with genes extending over long genomic regions (“large genes”) [5]. The *FHIT* gene in FRA3B and *WWOX* in FRA16D are striking examples, measuring approximately 1.5 and 1.1 Mb respectively, compared with a mean of 10–15 kb for protein coding genes. The *PARK2* gene, at approximately 1.4 Mb is also associated with FRA6E and may be down-regulated in ovarian tumors [30]. Intriguingly, genes over 800 kb may be prone to form RNA:DNA hybrid loops (termed R-loops) at sites of replication–transcription collision [35]. R-loops are structures formed by the association of the nascent transcript with the DNA template strand leaving unpaired the complementary non-coding DNA strand. Replication of large genes is time consuming and exposes the replication machinery to a risk of collision with the transcriptional machinery. In such an occurrence, the elongating RNA polymerase is blocked, leading to increased R-loop formation at Pol II pause sites. As a result, collision events may induce CFS breakage and a consequent enhancement of genomic instability [35].

Recent data have shed new light on the dynamics of the replication process at CFSs, providing several new mechanistic aspects explaining their instability (reviewed in [13, 14]). In the first one, it was shown that stability of FRA16C is perturbed under RS, as RFs progress more slowly and stall upon accounting AT-rich regions within this site. While in the bulk genome dormant origins are activated to complete replication, these are not available within FRA16C leading to delayed replication and instability [14] (also see B. Kerem chapter in this issue). A second report studying the mechanistic fragility of FRA3B, showed that fork speed slowing and stalling is similar with respect to the bulk genome, even under RS [26] (also see M. Debatisse chapter in this issue). In this case, the inability to complete replication was attributed to a large 700-kb core within this site that was found to be poor in origins. To accomplish replication of this site, origins from a long distance, located in the flanking regions, are required to come in and cover its length. The density and timing of origin firing events in the flanking regions seem to dictate the timing of FRA3B replication completion, thus influencing its stability. In a third mechanistic model regarding the FRA6E site, both replication arrest and paucity of origin activation lead to RS sensitivity, providing a functional combination of the two previous models [36]. Interestingly, some CFSs like the FRA3B do not exhibit stably these replication features in each cell type, suggesting that CFSs may demonstrate different patterns of instability, which are tissue specific. This may also explain why in various malignancies distinct profiles of GI are observed at CFSs that characterize each type of cancer. It would be interesting in the future to define the replication behavior of each CFS according to the specific cell type. That would be helpful in defining and possibly predicting the precise patterns of GI that take place during cancer development.

The replication density and timing of the genome has been proposed to be highly flexible and epigenetically controlled rather than directed by specific sequence motifs [13]. Therefore, an epigenetic control of CFSs replication stability may also apply. In support of this is the observed H3K9/14 hypoacetylation pattern displayed by the six most expressed CFSs in lymphoblastoid cells [9]. This histone modification has been reported to be associated with chromatin compactness and increased breakage. Also, regions with evenly spaced nucleosomes, an unusual chromatin structure preferentially formed at promoters and regulatory binding sites, have also been observed in FRA3B [37].

Overall, it seems that there is no single mechanism that can explain the fragility of CFSs but rather a multitude. They depend on several characteristics, including structural properties of the FSs as well as dynamic features governing their replication that apply in a given cell type. Interestingly, they are not necessarily mutually exclusive and often can function in complementary ways [13, 14]. The only common shared aspect by all these mechanisms is that they can eventually lead to a mechanical breakage of CFSs.

## Maintenance of fragile site integrity

Instability at FSs is a recognized signature of DNA damage induced by replication stress [2] and it is detected from the earliest premalignant stages [3, 4, 19]. Replication checkpoints are activated in response to the stress induced at CFSs [38]. Central to these checkpoints are the DNA damage response kinases, ATM and ATR, which respectively sense DNA double-strand breaks and RF integrity [38]. Specific targeting of these kinases in cellular models revealed that ATR disruption or hypomorphic mutations lead to chromosomal instability within CFS even under normal replication, a phenomenon that is aggravated after low doses of APH [39]. In addition, dual inhibition of ATM and ATR using caffeine has been found to significantly increase CFS breakage compared with ATR deficiency alone, denoting also a role for ATM and a possible interplay with ATR in CFS protection [40, 41]. Inactivation of several down-stream components of the ATR network like Chk1, HUS1, Claspin, and SMC1 revealed similar effects, although not as efficient as ATR loss (Table 1) [31, 42–44].

**Table 1:** Factors involved in control of CFSs stability and genome integrity

Factor	Function	Reference
ATR	Main kinase-activating replication checkpoint	[39, 75]
ATM	Complementary acting kinase	[76]
Chk1	Main ATR-downstream kinase involved in activation of the replication checkpoint	[31]
Chk2	ATM-downstream kinase	[76]
HUS1	Participates in the Rad9-Rad1-Hus1 (9-1-1) complex and is homologous to the PCNA clamp. The complex phosphorylates ATR substrates upon loading to sites of DNA damage	[42]
Claspin	Encodes for an adaptor protein which binds to BRCA1 and Chk1 and facilitates the ATR-dependent phosphorylation of both proteins during DNA replication stress in human cells	[43]
SMC1	Member of the family of “structural maintenance of chromosomes” proteins participating in chromosome condensation, sister chromatid cohesion, and DNA repair. Prevents the collapse of stalled replication fork in an ATR-dependent manner and is required for S-phase checkpoint activation	[77]
BRCA1	ATR substrate, implicated in the activation of the G2/M checkpoint, homologous recombination, and DSB repair	[78]
FANCD2	Component of the Fanconi anemia (FA) pathway, phosphorylated by ATR. Plays a role not only in HR-dependent replication recovery, but also in regulating CFSs stability	[79]
Polymerase η	Involved in DNA synthesis of complex sequences, like repetitive and secondary structure that impede replication performing, the so-called ‘by-pass’ function	[80]
Rev3	Catalytic subunit of Polζ that is required for maintaining fragile site stability in human cells	[81]
Polymerase κ	Involved in DNA synthesis of complex sequences, like repetitive and secondary structure that impede replication performing	[82]
WRN	RecQ helicase regulated in an ATR and ATM-dependent manner, which prevents DSBs formation at perturbed forks after replication stress. Promotes stability of arrested RFs and their efficient restart	[83]
BLM	RecQ helicase contributing to restarting stalled forks through unwinding DNA structures and/or homologous recombination, to maintenance of pyrimidine pools balance, regulation of fork speed and decatenation of UFBs at CFSs	[49, 50]
RECQ1	Member of the RecQ family of DNA helicases. Promotes fork recovery and repair	[84]
Topoisomerase I and II	Alleviate DNA secondary structures during replication by cleavage and re-ligation. Can facilitate oncogenic rearrangements at induced CFSs	[85]
MUS81-EME1	Endonuclease involved in resolving HJ-dependent replication intermediates. Required for fork repair and resolving UFBs	[41, 50]
PICH	PICH (Plk1 interacting checkpoint) is a helicase/translocase involved in resolving UFBs in mitosis	[49]
SNM1B/APOLLO	A nuclease component of the FA pathway involved in homology-directed repair. Also facilitates DNA localization of FANCD2 and BRCA1	[86]
Rad51	Component of the HR pathway involved in DSB repair and HJ mediated RF restart	[47]
DNA-PKcs	NHEJ pathway component	[47]
Ligase IV	NHEJ pathway component	[47]

These checkpoints are vital as they ensure that DNA is replicated and chromosomes are prepared for mitosis [38]. Nevertheless, CFSs exhibit an increased vulnerability to RS leading to the activation of repair mechanisms [31]. The frequently observed presence of sister chromatid exchanges (SCEs) at the majority of CFSs breaks after APH treatment suggests that homologous recombination (HR) plays a major role in response to DSBs induced under conditions of RS [45]. The FANCD2 component of the Fanconi anemia (FA) pathway has been shown to play a role not only in HR-dependent replication recovery, but also in regulating CFSs stability (Table 1) (also reviewed in [31]). Similarly, activation of BRCA1 and other DSB repair proteins like RAD51 have also been found to be vital for maintaining CFSs stability (Table 1) [46]. Apart from HR the non-homologous end join pathway is also essential for chromosomal stability at these sites [47]. Specifically, by knocking down Rad51, DNA-PKcs, or Ligase IV, a significantly increased expression of CFSs under RS has been demonstrated. Notably, MDC1 and γ-H2AX foci were formed and co-localized with those of Rad51 and DNA-PKcs, while γ-H2AX and phospho-DNA-PKcs foci localized at expressed FSs on metaphase chromosomes.

Other components implicated in resolving replication over specific CFSs region include specialized polymerases (Table 1) [48]. These polymerases, like DNA polymerase eta (Pol η), mainly deal with DNA synthesis of complex sequences, like repetitive and secondary structures that impede replication, by performing the so-called ‘by-pass’ function. Depletion of these specialized polymerases has been shown to lead to persistence of unreplicated CFSs in mitosis. Various helicases/translocases have been proposed to promote fork restart at CFSs in non-redundant ways, like the BLM, WRN, and RECQ1 through Holliday junction-mediated fork remodeling that is independent of DSB formation (Table 1) [41]. Their main purpose seems to be resetting of structural intermediates arising from HR as well as unwinding of DNA secondary structures, in order to facilitate replication fork restart. Alternatively, nucleases, such as the structural endonuclease MUS81-EME1 and the FA pathway nuclease SNM1B/APOLLO, are responsible for DSB-mediated fork restart and/or elimination of permanently collapsed forks by cleavage of replication intermediates and consequent DNA synthesis (Table 1) [41].

Many of the above-described factors have been shown to stabilize CFSs during S-phase replication. Nevertheless, recent observations have shown that under RS, non-fully replicated or interlinked DNA at CFSs may escape S and G2–M checkpoints and enter mitosis (reviewed in [13, 49, 50]). Attempts to segregate these intermediates lead to sister chromatid entanglement followed by non-disjunction, ultimately leading to formation of ultra-fine bridges (UFBs) in anaphase cells. UFBs are defined by FANCD2/FANCI FA proteins binding to their edges, while BLM and PICH (Plk1-interacting checkpoint helicase) attach along the bridge. These persistent replication intermediates have been shown to be processed by the MUS81-EME1 nuclease in early M-phase, possibly with the help of ERCC1, to provide a controlled production of DNA breaks, aiming to allow undisturbed disjunction of sister chromatids. In case of failure, UFBs are formed as mentioned during anaphase. At this stage, BLM helicase and PICH translocase assisted by topoisomerase IIIa (TOPIIIa) and the BLM-associated proteins RMI1/2 function as a second line of defense by decatenating these structures and permitting chromatid segregation [50]. If unresolved UFBs still persist, they will eventually lead to chromosome miss-segregation by uneven distribution of DNA between the daughter cells and micronuclei formation. The transmitted errors at CFSs will be shielded in 53BP1 nuclear bodies in the emerging G1-daughter cells and possibly replicated in the S-phase by high-fidelity polymerases. These results pose a new light on CFSs cleavage, and led to the proposal that apart from being detrimental in initiating genomic instability, it can also serve as a mechanism for controlled production of DNA breaks that rather maintain than compromise genome integrity.

Several questions though emerge from this model that has been established with extrinsic factors (chemical inhibitors) that induce RS. How does this model apply and/or differentiate pre-malignant cells that are known to undergo OIRS? Which of the two types of damage, MUS81-EME1 cleavage or the decatenation inability, confers to the cancer-associated genome instability? A tempting but speculative model is that during premalignant stages, MUS81-EME1 cleavage activity is probably aberrantly increased, leading to a high frequency of DSBs, particularly at CFSs. This could be mediated by the active oncogenes that are present at such stages [2] and which in turn increase the activity of CDKs that regulate MUS81-EME1 expression [50]. As long as the checkpoints and their p53 effector are intact, the damage is repaired or the antitumor barriers of apoptosis and senescence eliminate such cells [2]. A similar scenario regarding gross genome damage due to decatenation inability may apply. Notably, it has been recently shown that under moderate RS, CFSs breaks can escape from efficient ATR checkpoint surveillance, leading to mitotic tolerance of such aberrations [44]. Such a pool of cells could undergo selection for loss of checkpoint function(s) and eventually accumulate DNA damage, probably through both MUS81-EME1 processing and chromatid non-disjunctions, provided that they are compatible with survival. Eventually, progression to full malignancy will ensue. This scenario fully concurs with our previous findings showing a prevalence of CFSs breakage along with the presence of UFBs and micronuclei in U2OS cells experiencing OIRS due to sustained expression of the replication licensing factor (RLF) Cdt1 [51]. Importantly, clones of these cells that “escaped” from the antitumor barriers, after prolonged Cdt1 expression, acquired a highly invasive potential. In a paradoxical way, this recently described model of deliberate CFSs controlled breakage to protect genome integrity of cells, may apply to malignant cells in the sense that it allows them to survive at the expense of genome integrity. Further expanding on this model, an emerging question concerns the effect exerted from the CFSs’ instability on the various elements like genes and non-coding RNAs that are located within them.

## Functional elements in fragile sites

Several major publications arising from the ENCODE project [10] have underlined the importance of non-coding DNA. Non-coding regions of the genome have been found to participate in biochemical reactions with regulatory potential, such as transcription factor binding, epigenetic modifications, or long-distance interactions. Numerous functions have been attributed to non-coding RNA, including the expanding and best understood family of microRNAs (miRs) that are involved in post-transcriptional regulation of messenger RNA [52]. As a result of this progress, CFSs content may be now understood at a finer scale and the implications of CFS breakage will have to be reexamined carefully.

### Fragile sites and cancer-associated genes

**Fig. 2:**
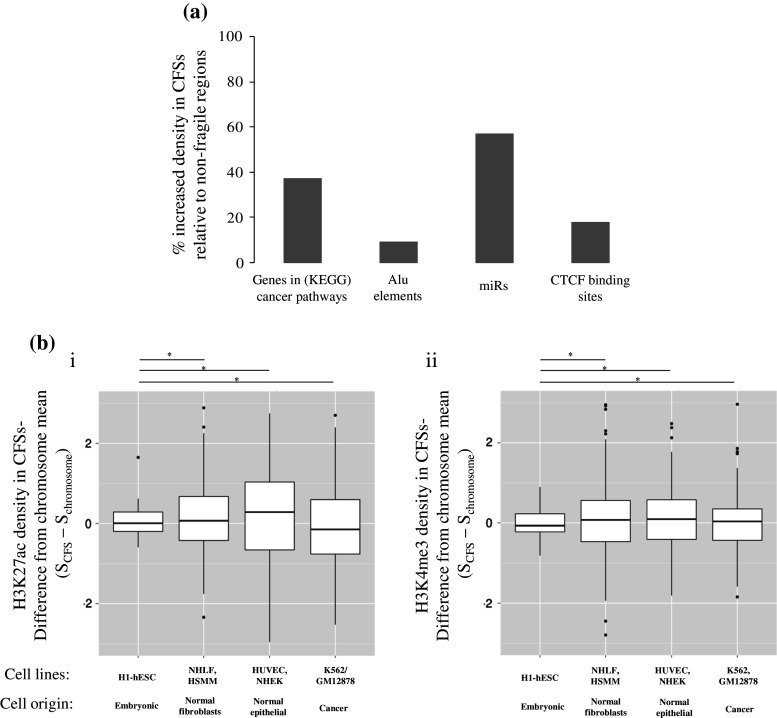

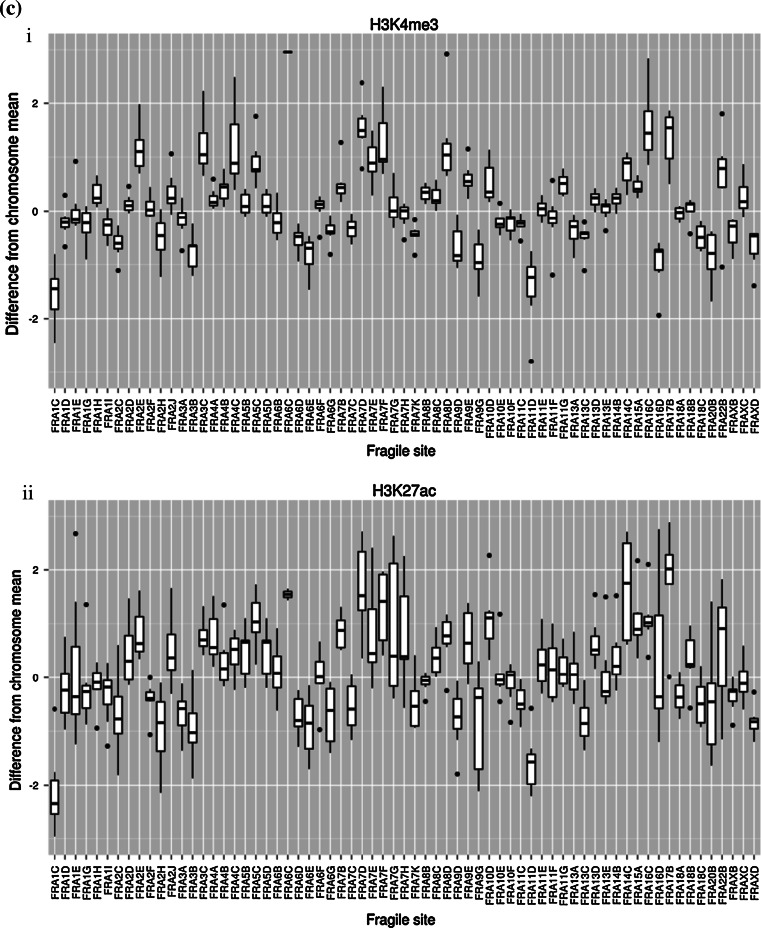
Frequency of cancer-related genes, repetitive elements, miRs, binding elements, and histone marks in CFSs. **a** CFSs exhibit a higher density of cancer-related genes (obtained from Kyoto Encyclopedia of Genes and Genomes), Alu repetitive elements [19], miRs, and the CTCF binding element relative to non-fragile regions. **b** CFSs exhibit a differential density of the histone marks (i) Histone 3 lysine 27 acetylation (H3K27ac) and (ii) Histone 3 lysine 4 trimethylation (H3K4me3), relative to non-fragile regions that is cell type origin-dependent (data concerning histone modifications derived from ChIP-seq experiments belonging to the ENCODE project were downloaded from the UCSC server (http://hgdownload.cse.ucsc.edu/goldenPath/hg19/encodeDCC/wgEncodeRegMarkH3k4me3/, http://hgdownload.cse.ucsc.edu/goldenPath/hg19/encodeDCC/wgEncodeRegMarkH3k27ac/). Specifically, we obtained bigWig files for H3k4me3 and H3k27ac modifications in the GM12878, H1-hesc, HSMM, HUVEC, K562, NHEK, and NHLF cell types. Information concerning regions of interest was extracted with the bigWigSummary utility, also available from the UCSC server. Specifically, the average signal was calculated for every chromosome in every cell line by repeatedly invoking: bigWigSummary-type = mean “bigwigfile” chrN start end. Similarly, the average signal was calculated for cytogenetically and molecularly mapped fragile sites. For every defined fragile site, the mean histone modification signal of the corresponding chromosome was subtracted from the mean signal of the fragile region. Signal difference from mean for site *i* = mean(FS_*i*_) − mean(chromosome_FS*i*_). The chromosome means varied within each cell type (data not shown), as did the histone modifications between cell types) (*S* histone signal). **c** Frequency of histone marks per CFSs. Each CFS exhibits a differential density of the histone marks. (i) Histone 3 lysine 4 trimethylation (H3K4me3) and (ii) Histone 3 lysine 27 acetylation (H3K27ac), relative to non-fragile regions averaged over all cell types presented in Fig. 2 (using the data generated for Fig. 2b, we also plotted a *boxplot* for individual cytogenetically defined CFSs in all 7 cell lines mentioned above with respect to H3K4me3 and H3K27ac. Significant heterogeneity between CFSs can be observed)

While available data point to an overlap between genes and CFSs [5], there is only one report showing that CFSs are denser in protein coding genes, with their distribution among fragile versus non-fragile regions varying among the chromosomes [7]. At the same time, a systematic review for the density of genes present within these genome areas is not available. To address this question, we retrieved a list of 327 genes participating in pathways in cancer from the Kyoto Encyclopedia of Genes and Genomes (KEGG)^1^ and investigated their association with both cytogenetically and molecularly mapped CFSs. We found that 110 cancer-related genes (33.6 % of all cancer-related genes) are located within CFSs (Table 2). Based on this, the density of cancer-related genes in a cytogenetically defined CFS compared to the rest of the genome is 37.2 % higher (Fig. 2a).

**Table 2:** Cytogenetic defined common fragile sites (CFSs) association with cancer genes and miRs

FRA	Chr	Start	End	Band	Type	Frequency	Cancer genes	miRNAs	Reference
FRA1A	1	1	2,8000,000	1p36	Aphidicolin	Common	DVL1, PIK3CD, MTOR, CASP9, CDC42, WNT4, E2F2	hsa-mir-6859-1, hsa-mir-1302-2, hsa-mir-6723, hsa-mir-200b, hsa-mir-200a, hsa-mir-429, hsa-mir-6726, hsa-mir-6727, hsa-mir-6808, hsa-mir-4251, hsa-mir-551a, hsa-mir-4417, hsa-mir-4689, hsa-mir-4252, hsa-mir-6728, hsa-mir-34a, hsa-mir-5697, hsa-mir-1273d, hsa-mir-6729, hsa-mir-7846, hsa-mir-4632, hsa-mir-6730, hsa-mir-3675, hsa-mir-3972, hsa-mir-4695, hsa-mir-1290, hsa-mir-6084, hsa-mir-1256, hsa-mir-4418, hsa-mir-6127, hsa-mir-4684, hsa-mir-4253, hsa-mir-3115, hsa-mir-4419a, hsa-mir-378f, hsa-mir-6731, hsa-mir-4425, hsa-mir-3917, hsa-mir-1976	[90]
FRA1B	1	5,0700,001	6,1300,000	1p32	Aphidicolin	Common	JUN	hsa-mir-4421, hsa-mir-6500, hsa-mir-761, hsa-mir-1273f, hsa-mir-5095, hsa-mir-1273g, hsa-mir-4781, hsa-mir-4422, hsa-mir-4711	[8]
FRA1C	1	68,900,001	69,700,000	1p31.2	Aphidicolin	Common			
FRA1D	1	84,900,001	94,700,000	1p22	Aphidicolin	Common		hsa-mir-4423, hsa-mir-7856, hsa-mir-760	[91]
FRA1E	1	99,700,001	102,200,000	1p21.2	Aphidicolin	Common		hsa-mir-553	[92]
FRA1F	1	142,600,001	155,000,000	1q21	Aphidicolin	Common	ARNT, TPM3, CKS1B	hsa-mir-3118-1, hsa-mir-3118-2, hsa-mir-3118-3, hsa-mir-6077-1, hsa-mir-6736, hsa-mir-5087, hsa-mir-6077-2, hsa-mir-6878, hsa-mir-4257, hsa-mir-554, hsa-mir-8083, hsa-mir-6737, hsa-mir-5698, hsa-mir-190b, hsa-mir-4258	[91]
FRA1G	1	172,900,001	176,000,000	1q25.1	Aphidicolin	Common			
FRA1H	1	224,100,001	236,600,000	1q42	5-Azacytidine	Common	WNT9A, WNT3A, EGLN1	hsa-mir-320b-2, hsa-mir-4742, hsa-mir-6741, hsa-mir-5008, hsa-mir-3620, hsa-mir-6742, hsa-mir-4666a, hsa-mir-1182, hsa-mir-4427, hsa-mir-4671, hsa-mir-4753, hsa-mir-1537	[93]
FRA1I	1	243,700,001	249,250,621	1q44	Aphidicolin	Common		hsa-mir-3916, hsa-mir-3124	
FRA1J	1	128,900,001	142,600,000	1q12	5-Azacytidine	Common			[94]
FRA1K	1	185,800,001	198,700,000	1q31	Aphidicolin	Common	TPR, PTGS2	hsa-mir-4426, hsa-mir-1278, hsa-mir-4735	[95]
FRA1L	1	61,300,001	84,900,000	1p31	Aphidicolin	Common	JAK1	hsa-mir-3116-1, hsa-mir-3116-2, hsa-mir-6068, hsa-mir-4794, hsa-mir-3671, hsa-mir-101-1, hsa-mir-3117, hsa-mir-1262, hsa-mir-186, hsa-mir-7156	
FRA1M	1	94,700,001	99,700,000	1p21.3	Folic acid	Rare		hsa-mir-378g, hsa-mir-2682, hsa-mir-137	
FRA2A	2	96,800,001	102,700,000	2q11.2	Folic acid	Rare		hsa-mir-3127, hsa-mir-5696	
FRA2B	2	110,200,001	114,400,000	2q13	Folic acid	Rare	PAX8	hsa-mir-4267, hsa-mir-4436b-1, hsa-mir-4436b-2, hsa-mir-4435-2, hsa-mir-4771-2, hsa-mir-1302-3	[96]
FRA2C	2	16,700,001	19,200,000	2p24.2	Aphidicolin	Common			[97]
FRA2D	2	52,900,001	55,000,000	2p16.2	Aphidicolin	Common		hsa-mir-4431, hsa-mir-3682	[27]
FRA2E	2	68,600,001	75,000,000	2p13	Aphidicolin	Common	TGFA	hsa-mir-3126, hsa-mir-1285-2	[98]
FRA2F	2	135,100,001	136,800,000	2q21.3	Aphidicolin	Common		hsa-mir-5590, hsa-mir-128-1	[8]
FRA2G	2	169,700,001	18,3000,000	2q31	Aphidicolin	Common	ITGA6	hsa-mir-933, hsa-mir-10b, hsa-mir-7704, hsa-mir-1246, hsa-mir-4444-1, hsa-mir-3128, hsa-mir-6512, hsa-mir-1258, hsa-mir-4437	[99, 100]
FRA2H	2	183,000,001	189,400,000	2q32.1	Aphidicolin	Common	ITGAV	hsa-mir-548ae-1, hsa-mir-561	[100]
FRA2I	2	197,400,001	209,000,000	2q33	Aphidicolin	Common	CASP8, FZD7, FZD5	hsa-mir-3130-2, hsa-mir-3130-1, hsa-mir-2355, hsa-mir-7845, hsa-mir-1302-4, hsa-mir-4775	[101]
FRA2J	2	237,300,001	243,199,373	2q37.3	Aphidicolin	Common		hsa-mir-6811, hsa-mir-4440, hsa-mir-4441, hsa-mir-4269, hsa-mir-2467, hsa-mir-4786, hsa-mir-149, hsa-mir-3133	[102]
FRA2K	2	144,100,001	148,700,000	2q22.3	Folic acid	Rare			[103]
FRA2L	2	83,300,001	90,500,000	2p11.2	Folic acid		TCF7L1	hsa-mir-6071, hsa-mir-4779, hsa-mir-4771-1, hsa-mir-4435-1, hsa-mir-4780, hsa-mir-4436a	[104]
FRA2S	2	144,100,001	154,900,000	2q22.3-2q23.3	Aphidicolin	Common		hsa-mir-4773-2, hsa-mir-4773-1	[105]
FRA3A	3	23,900,001	26,400,000	3p24.2	Aphidicolin	Common	RARB	hsa-mir-4792, hsa-mir-4442	
FRA3B	3	58,600,001	63,700,000	3p14.2	Aphidicolin	Common			[106]
FRA3C	3	182,700,001	187,900,000	3q27	Aphidicolin	Common	DVL3	hsa-mir-4448, hsa-mir-1224, hsa-mir-5588, hsa-mir-548aq, hsa-mir-1248	[107]
FRA3D	3	148,900,001	160,700,000	3q25	Aphidicolin	Common		hsa-mir-5186, hsa-mir-3919, hsa-mir-15b, hsa-mir-16-2	[107]
FRA4A	4	6,000,001	11,300,000	4p16.1	Aphidicolin	Common		hsa-mir-4798, hsa-mir-4274, hsa-mir-95, hsa-mir-548i-2, hsa-mir-3138	
FRA4B	4	527,00,001	59,500,000	4q12	BrdU	Common	PDGFRA, KIT	hsa-mir-4449	
FRA4C	4	139,500,001	141,500,000	4q31.1	Aphidicolin	Common			[108]
FRA4D	4	11,300,001	35,800,000	4p15	Aphidicolin	Common		hsa-mir-572, hsa-mir-5091, hsa-mir-218-1, hsa-mir-7978, hsa-mir-573, hsa-mir-4275	
FRA4F	4	88,000,001	98,800,000	4q22	Aphidicolin	Common		hsa-mir-5705	[109]
FRA5A	5	28,900,001	42,500,000	5p13	BrdU	Common	SKP2	hsa-mir-4279, hsa-mir-579, hsa-mir-580, hsa-mir-3650	
FRA5B	5	92,300,001	98,200,000	5q15	BrdU	Common		hsa-mir-2277, hsa-mir-583	
FRA5C	5	130,600,001	136,200,000	5q31.1	Aphidicolin	Common	TCF7	hsa-mir-6830, hsa-mir-3936, hsa-mir-1289-2, hsa-mir-3661, hsa-mir-4461, hsa-mir-5692c-1	[110]
FRA5D	5	92,300,001	98,200,000	5q15	Aphidicolin	Common		hsa-mir-2277, hsa-mir-583	
FRA5E	5	18,400,001	28,900,000	5p14	Aphidicolin	Common			[95]
FRA5F	5	98,200,001	109,600,000	5q21	Aphidicolin	Common		hsa-mir-548p	
FRA5G	5	168,500,001	180,915,260	5q35	Folic acid	Rare	FGF18, MAPK9	hsa-mir-585, hsa-mir-378e, hsa-mir-3912, hsa-mir-5003, hsa-mir-8056, hsa-mir-4634, hsa-mir-1271, hsa-mir-4281, hsa-mir-1229, hsa-mir-340, hsa-mir-8089, hsa-mir-4638	
FRA6A	6	13,400,001	15,200,000	6p23	Folic acid	Rare			[111]
FRA6B	6	4,200,001	71,00000	6p25.1	Aphidicolin	Common		hsa-mir-3691, hsa-mir-7853, hsa-mir-5683	
FRA6C	6	25,200,001	27,000,000	6p22.2	Aphidicolin	Common			[112]
FRA6D	6	70,000,001	75,900,000	6q13	BrdU	Common		hsa-mir-30c-2, hsa-mir-30a, hsa-mir-4282	
FRA6E	6	16,1000,001	164,500,000	6q26	Aphidicolin	Common			[113]
FRA6F	6	105,500,001	114,600,000	6q21	Aphidicolin	Common	LAMA4, HDAC2	hsa-mir-587	[114]
FRA6G	6	88,000,001	93,100,000	6q15	Aphidicolin	Common		hsa-mir-4464, hsa-mir-4643	
FRA6H	6	30,400,001	46,200,000	6p21	Aphidicolin	Common	RXRB, PPARD, CDKN1A, VEGFA, HSP90AB1	hsa-mir-877, hsa-mir-4640, hsa-mir-6891, hsa-mir-6832, hsa-mir-4646, hsa-mir-1236, hsa-mir-6721, hsa-mir-6833, hsa-mir-3135b, hsa-mir-219a-1, hsa-mir-6873, hsa-mir-6834, hsa-mir-5004, hsa-mir-3934, hsa-mir-7159, hsa-mir-1275, hsa-mir-6835, hsa-mir-7111, hsa-mir-5690, hsa-mir-3925, hsa-mir-4462, hsa-mir-4641, hsa-mir-6780b, hsa-mir-4647, hsa-mir-4642, hsa-mir-586	[115]
FRA7A	7	54,000,001	58,000,000	7p11.2	Folic acid	Rare	EGFR	hsa-mir-4283-1, hsa-mir-3147	
FRA7B	7	1	7,300,000	7p22	Aphidicolin	Common	PDGFA, RAC1	hsa-mir-339, hsa-mir-4655, hsa-mir-6836, hsa-mir-4648, hsa-mir-4656, hsa-mir-589, hsa-mir-6874, hsa-mir-3683	[116]
FRA7C	7	35,000,001	37,200,000	7p14.2	Aphidicolin	Common		hsa-mir-1200	
FRA7D	7	43,300,001	45,400,000	7p13	Aphidicolin	Common		hsa-mir-6837, hsa-mir-6838, hsa-mir-4649, hsa-mir-4657	
FRA7E	7	91,100,001	92,800,000	7q21.2	Aphidicolin	Common	CDK6	hsa-mir-1285-1	[117]
FRA7F	7	98,000,001	107,400,000	7q22	Aphidicolin	Common	PIK3CG	hsa-mir-3609, hsa-mir-25, hsa-mir-93, hsa-mir-106b, hsa-mir-4658, hsa-mir-6840, hsa-mir-6875, hsa-mir-4653, hsa-mir-4285, hsa-mir-548o, hsa-mir-5090, hsa-mir-4467	
FRA7G	7	114,600,001	117,400,000	7q31.2	Aphidicolin	Common	MET, WNT2	hsa-mir-6132	[118]
FRA7H	7	130,400,001	132,600,000	7q32.3	Aphidicolin	Common		hsa-mir-29a, hsa-mir-29b-1	[119]
FRA7I	7	147,900,001	159,138,663	7q36	Aphidicolin	Common	SHH	hsa-mir-671, hsa-mir-3907, hsa-mir-153-2, hsa-mir-595, hsa-mir-5707	[120]
FRA7J	7	59,900,001	77,500,000	7q11	Aphidicolin	Common	FZD9	hsa-mir-4283-2, hsa-mir-6839, hsa-mir-4650-1, hsa-mir-3914-1, hsa-mir-3914-2, hsa-mir-4650-2, hsa-mir-4284, hsa-mir-590, hsa-mir-4651	
FRA7K	7	107,400,001	114,600,000	7q31.1	Aphidicolin	Common	LAMB1, LAMB4	hsa-mir-3666	[121]
FRA8A	8	101,600,001	106,200,000	8q22.3	Folic acid	Rare	FZD6	hsa-mir-7705, hsa-mir-5680, hsa-mir-3151, hsa-mir-548a-3	
FRA8B	8	93,300,001	99,000,000	8q22.1	Aphidicolin	Common	CCNE2	hsa-mir-8084, hsa-mir-378d-2, hsa-mir-3150b, hsa-mir-3150a	
FRA8C	8	117,700,001	127,300,000	8q24.1	Aphidicolin	Common		hsa-mir-3610, hsa-mir-548az, hsa-mir-4663, hsa-mir-548aa-1, hsa-mir-548d-1, hsa-mir-6844, hsa-mir-4662b, hsa-mir-4662a	[91]
FRA8D	8	139,900,001	146364,022	8q24.3	Aphidicolin	Common	PTK2	hsa-mir-151a, hsa-mir-1302-7, hsa-mir-4472-1, hsa-mir-4664, hsa-mir-937, hsa-mir-6845, hsa-mir-661, hsa-mir-6846, hsa-mir-6847, hsa-mir-7112-1, hsa-mir-7112-2, hsa-mir-6848, hsa-mir-939, hsa-mir-1234, hsa-mir-6849, hsa-mir-6893, hsa-mir-6850	[66]
FRA8E	8	117,700,001	127,300,000	8q24.1	Distamycin A	Rare		hsa-mir-3610, hsa-mir-548az, hsa-mir-4663, hsa-mir-548aa-1, hsa-mir-548d-1, hsa-mir-6844, hsa-mir-4662b, hsa-mir-4662a	[122]
FRA9A	9	19,900,001	33,200,000	9p21	Folic acid	Rare	CDKN2A, CDKN2B	hsa-mir-4473, hsa-mir-4474, hsa-mir-491, hsa-mir-31, hsa-mir-876, hsa-mir-873	[123]
FRA9B	9	114,900,001	117,700,000	9q32	Folic acid	Rare		hsa-mir-455	
FRA9C	9	19,900,001	33,200,000	9p21	BrdU	Common	CDKN2A, CDKN2B	hsa-mir-4473, hsa-mir-4474, hsa-mir-491, hsa-mir-31, hsa-mir-876, hsa-mir-873	
FRA9D	9	90,400,001	91,800,000	9q22.1	Aphidicolin	Common		hsa-mir-4289	[91]
FRA9E	9	114,900,001	117,700,000	9q32	Aphidicolin	Common		hsa-mir-455	[124]
FRA9F	9	50,700,001	65,900,000	9q12	5-Azacytidine	Common			
FRA9G	9	16,600,001	18,500,000	9p22.2	Aphidicolin	Common			[125]
FRA10A	10	89,500,001	97,000,000	10q23.3	Folic acid	Rare	PTEN, FAS	hsa-mir-4679-2, hsa-mir-4679-1, hsa-mir-107	[126]
	10	99,300,001	101,900,000	10q24.2				hsa-mir-1287, hsa-mir-4685, hsa-mir-6507	
FRA10AC1	10	89,500,001	97,000,000	10q23.3	Folic acid	Rare	PTEN, FAS	hsa-mir-4679-2, hsa-mir-4679-1, hsa-mir-107	[127]
	10	99,300,001	101,900,000	10q24.2				hsa-mir-1287, hsa-mir-4685, hsa-mir-6507	
FRA10B	10	111,900,001	114,900,000	10q25.2	BrdU	Rare		hsa-mir-4680, hsa-mir-548e, hsa-mir-6715b, hsa-mir-6715a, hsa-mir-4295	[128]
FRA10C	10	52,900,001	70,600,000	10q21	BrdU	Common	CCDC6, CTNNA3	hsa-mir-605, hsa-mir-548f-1, hsa-mir-3924, hsa-mir-1296, hsa-mir-7151, hsa-mir-1254-1	[129]
FRA10D	10	70,600,001	74,900,000	10q22.1	Aphidicolin	Common		hsa-mir-7152, hsa-mir-4676	[126]
FRA10E	10	111,900,001	114,900,000	10q25.2	Aphidicolin	Common		hsa-mir-4680, hsa-mir-548e, hsa-mir-6715b, hsa-mir-6715a, hsa-mir-4295	[126]
FRA10F	10	119,100,001	127,500,000	10q26.1	Aphidicolin	Common	FGFR2, CTBP2	hsa-mir-4681, hsa-mir-4682, hsa-mir-3941, hsa-mir-4296	[130]
FRA10G	10	42,300,001	52,900,000	10q11.2	Aphidicolin	Common	RET, MAPK8, NCOA4	hsa-mir-5100, hsa-mir-3156-1, hsa-mir-4294	[129]
FRA11A	11	68,400,001	70,400,000	11q13.3	Folic acid	Rare	CCND1, FGF19, FGF4, FGF3, FADD	hsa-mir-3164, hsa-mir-548k	[131]
FRA11B	11	114,500,001	121,200,000	11q23.3	Folic acid	Rare	CBL	hsa-mir-6716, hsa-mir-4492, hsa-mir-3656, hsa-mir-6756	[132, 133]
FRA11C	11	16,200,001	21,700,000	11p15.1	Aphidicolin	Common		hsa-mir-3159, hsa-mir-4486, hsa-mir-4694	[91]
FRA11D	11	26,100,001	27,200,000	11p14.2	Aphidicolin	Common			
FRA11E	11	31,000,001	36,400,000	11p13	Aphidicolin	Common		hsa-mir-1343, hsa-mir-3973	[134]
FRA11F	11	85,600,001	88,300,000	11q14.2	Aphidicolin	Common	FZD4	hsa-mir-6755, hsa-mir-3166	[135]
FRA11G	11	114,500,001	121,200,000	11q23.3	Aphidicolin	Common	CBL	hsa-mir-6716, hsa-mir-4492, hsa-mir-3656, hsa-mir-6756	[133]
FRA11H	11	63,400,001	77,100,000	11q13	Aphidicolin	Common	VEGFB, BAD, RELA, GSTP1, CCND1, FGF19, FGF4, FGF3, FADD, WNT11	hsa-mir-7155, hsa-mir-1237, hsa-mir-192, hsa-mir-194-2, hsa-mir-6750, hsa-mir-6749, hsa-mir-6879, hsa-mir-6751, hsa-mir-612, hsa-mir-4690, hsa-mir-4489, hsa-mir-3163, hsa-mir-6860, hsa-mir-6752, hsa-mir-7113, hsa-mir-4691, hsa-mir-6753, hsa-mir-3164, hsa-mir-548k, hsa-mir-3664, hsa-mir-6754, hsa-mir-3165, hsa-mir-139, hsa-mir-4692, hsa-mir-548al, hsa-mir-4696, hsa-mir-326	[136]
FRA11I	11	16,200,001	21,700,000	11p15.1	Distamycin A	Rare		hsa-mir-3159, hsa-mir-4486, hsa-mir-4694	
FRA12A	12	46,400,001	54,900,000	12q13.1	Folic acid	Rare	WNT10B, WNT1	hsa-mir-4698, hsa-mir-4494, hsa-mir-6505, hsa-mir-1291, hsa-mir-4701, hsa-mir-1293, hsa-mir-6757, hsa-mir-196a-2, hsa-mir-615, hsa-mir-3198-2, hsa-mir-148b	[137]
FRA12B	12	80,300,001	92,600,000	12q21.3	Aphidicolin	Common	KITLG	hsa-mir-617, hsa-mir-618, hsa-mir-4699	
FRA12C	12	114,300,001	120,700,000	12q24.2	BrdU	Rare		hsa-mir-620, hsa-mir-4472-2, hsa-mir-1178, hsa-mir-4498	[138]
FRA12D	12	112,300,001	114,300,000	12q24.13	Folic acid	Rare		hsa-mir-3657, hsa-mir-6861, hsa-mir-1302-1, hsa-mir-7106, hsa-mir-6762	
FRA12E	12	109,000,001	133,851,895	12q24	Aphidicolin	Common	FZD10	hsa-mir-4496, hsa-mir-619, hsa-mir-4497, hsa-mir-6760, hsa-mir-6761, hsa-mir-3657, hsa-mir-6861, hsa-mir-1302-1, hsa-mir-7106, hsa-mir-6762, hsa-mir-620, hsa-mir-4472-2, hsa-mir-1178, hsa-mir-4498, hsa-mir-4700, hsa-mir-7107, hsa-mir-4304, hsa-mir-8072, hsa-mir-3908, hsa-mir-6880, hsa-mir-5188, hsa-mir-4419b, hsa-mir-3612, hsa-mir-6763	
FRA13A	13	34,000,001	35,500,000	13q13.2	Aphidicolin	Common			[139]
FRA13B	13	55,300,001	73,300,000	13q21	BrdU	Common		hsa-mir-5007, hsa-mir-3169, hsa-mir-548x-2, hsa-mir-4704	
FRA13C	13	59,600,001	62,300,000	13q21.2	Aphidicolin	Common		hsa-mir-3169	[140]
FRA13D	13	95,000,001	101,700,000	13q32	Aphidicolin	Common		hsa-mir-4501, hsa-mir-3170, hsa-mir-623, hsa-mir-4306	
FRA13E	13	73,300,001	79,000,000	13q22	Aphidicolin	Common		hsa-mir-3665	[115]
FRA14B	14	58,100,001	67,900,000	14q23	Aphidicolin	Common	HIF1A, MAX	hsa-mir-5586, hsa-mir-548h-1, hsa-mir-7855, hsa-mir-4706, hsa-mir-4708, hsa-mir-625	
FRA14C	14	67,900,001	70,200,000	14q24.1	Aphidicolin	Common		hsa-mir-5694	
FRA15A	15	59,100,001	67,500,000	15q22	Aphidicolin	Common	DAPK2, MAP2K1, SMAD3	hsa-mir-2116, hsa-mir-8067, hsa-mir-6085, hsa-mir-190a, hsa-mir-422a, hsa-mir-1272, hsa-mir-4511, hsa-mir-4311, hsa-mir-4512	[141]
FRA16A	16	14,800,001	16,800,000	16p13.11	Folic acid	Rare		hsa-mir-3179-1, hsa-mir-3670-1, hsa-mir-3180-1, hsa-mir-6511a-1, hsa-mir-6770-1, hsa-mir-1972-1, hsa-mir-6511b-2, hsa-mir-3180-4, hsa-mir-6506, hsa-mir-484, hsa-mir-3179-2, hsa-mir-3670-2, hsa-mir-3180-2, hsa-mir-6511a-2, hsa-mir-6770-2, hsa-mir-6511a-3	[142]
FRA16B	16	66,700,001	70,800,000	16q22.1	Distamycin A	Rare	CDH1	hsa-mir-328, hsa-mir-6773, hsa-mir-1538, hsa-mir-140, hsa-mir-1972-2	[143]
FRA16C	16	66,700,001	70,800,000	16q22.1	Aphidicolin	Common	CDH1	hsa-mir-328, hsa-mir-6773, hsa-mir-1538, hsa-mir-140, hsa-mir-1972-2	[144]
FRA16D	16	79,200,001	81,700,000	16q23.2	Aphidicolin	Common		hsa-mir-4720, hsa-mir-7854, hsa-mir-6504	[143]
FRA16E	16	24,200,001	28,100,000	16p12.1	Distamycin A	Rare		hsa-mir-1273h, hsa-mir-548w	[145]
FRA17A	17	10,700,001	16,000,000	17p12	Distamycin A	Rare		hsa-mir-744, hsa-mir-1269b, hsa-mir-548h-3, hsa-mir-4731	[123]
FRA17B	17	57,600,001	58,300,000	17q23.1	Aphidicolin	Common		hsa-mir-21, hsa-mir-4737	[112]
FRA18A	18	32,700,001	37,200,000	18q12.2	Aphidicolin	Common		hsa-mir-3975, hsa-mir-187, hsa-mir-3929, hsa-mir-4318	[146]
FRA18B	18	53,800,001	61,600,000	18q21.3	Aphidicolin	Common	BCL2	hsa-mir-122, hsa-mir-3591	[147]
FRA18C	18	66,800,001	68,700,000	18q22.2	Aphidicolin	Common			[148]
FRA19A	19	32,400,001	59,128,983	19q13	5-Azacytidine	Common	CEBPA, AKT2, EGLN2, TGFB1, CBLC, FGF21, BAX, FLT3LG, KLK3, BIRC8, PRKCG	hsa-mir-6887, hsa-mir-5196, hsa-mir-4530, hsa-mir-6719, hsa-mir-641, hsa-mir-6796, hsa-mir-6797, hsa-mir-4323, hsa-mir-8077, hsa-mir-4531, hsa-mir-8085, hsa-mir-6088, hsa-mir-330, hsa-mir-642a, hsa-mir-642b, hsa-mir-769, hsa-mir-320e, hsa-mir-3190, hsa-mir-3191, hsa-mir-6798, hsa-mir-4324, hsa-mir-150, hsa-mir-5088, hsa-mir-6799, hsa-mir-6800, hsa-mir-4749, hsa-mir-4750, hsa-mir-4751, hsa-mir-8074, hsa-mir-99b, hsa-let-7e, hsa-mir-125a, hsa-mir-6801, hsa-mir-643, hsa-mir-512-1, hsa-mir-512-2, hsa-mir-1323, hsa-mir-498, hsa-mir-520e, hsa-mir-515-1, hsa-mir-519e, hsa-mir-520f, hsa-mir-515-2, hsa-mir-519c, hsa-mir-1283-1, hsa-mir-520a, hsa-mir-526b, hsa-mir-519b, hsa-mir-525, hsa-mir-523, hsa-mir-518f, hsa-mir-520b, hsa-mir-518b, hsa-mir-526a-1, hsa-mir-520c, hsa-mir-518c, hsa-mir-524, hsa-mir-517a, hsa-mir-519d, hsa-mir-521-2, hsa-mir-520d, hsa-mir-517b, hsa-mir-520g, hsa-mir-516b-2, hsa-mir-526a-2, hsa-mir-518e, hsa-mir-518a-1, hsa-mir-518d, hsa-mir-516b-1, hsa-mir-518a-2, hsa-mir-517c, hsa-mir-520h, hsa-mir-521-1, hsa-mir-522, hsa-mir-519a-1, hsa-mir-527, hsa-mir-516a-1, hsa-mir-1283-2, hsa-mir-516a-2, hsa-mir-519a-2, hsa-mir-371a, hsa-mir-371b, hsa-mir-372, hsa-mir-373, hsa-mir-935, hsa-mir-4752, hsa-mir-8061, hsa-mir-7975, hsa-mir-6804, hsa-mir-6802, hsa-mir-6803, hsa-mir-6805, hsa-mir-6806, hsa-mir-4754, hsa-mir-6807	
FRA19B	19	1	20,000,000	19p13	Folic acid	Rare	FGF22, APC2, DAPK3, MAP2K2, PIK3R2	hsa-mir-1302-10, hsa-mir-4745, hsa-mir-3187, hsa-mir-1909, hsa-mir-1227, hsa-mir-6789, hsa-mir-4321, hsa-mir-7108, hsa-mir-7850, hsa-mir-637, hsa-mir-4746, hsa-mir-7-3, hsa-mir-4747, hsa-mir-6885, hsa-mir-6790, hsa-mir-3940, hsa-mir-6791, hsa-mir-6792, hsa-mir-4999, hsa-mir-5589, hsa-mir-4322, hsa-mir-1181, hsa-mir-1238, hsa-mir-638, hsa-mir-4748, hsa-mir-199a-1, hsa-mir-6793, hsa-mir-6886, hsa-mir-7974, hsa-mir-5684, hsa-mir-6794, hsa-mir-5695, hsa-mir-6515, hsa-mir-24-2, hsa-mir-27a, hsa-mir-23a, hsa-mir-181c, hsa-mir-181d, hsa-mir-1199, hsa-mir-639, hsa-mir-6795, hsa-mir-1470, hsa-mir-3188, hsa-mir-3189, hsa-mir-640	
FRA20A	20	17,900,001	21,300,000	20p11.23	Folic acid	Rare		hsa-mir-3192	
FRA20B	20	920,0001	12,100,000	20p12.2	Aphidicolin	Common		hsa-mir-6870	
FRA22A	22	37,600,001	51,304,566	22q13	Folic acid	Rare	RAC2, PDGFB, RBX1, EP300, WNT7B	hsa-mir-658, hsa-mir-659, hsa-mir-6820, hsa-mir-4534, hsa-mir-4766, hsa-mir-1281, hsa-mir-6889, hsa-mir-33a, hsa-mir-378i, hsa-mir-1249, hsa-mir-4762, hsa-mir-3619, hsa-let-7a-3, hsa-mir-4763, hsa-let-7b, hsa-mir-3201, hsa-mir-4535, hsa-mir-3667, hsa-mir-6821	[149]
FRA22B	22	29,600,001	32,200,000	22q12.2	Aphidicolin	Common		hsa-mir-3653, hsa-mir-6818, hsa-mir-3200, hsa-mir-3928, hsa-mir-7109	
FRAXA	X	142,100,001	147,100,000	Xq27.3	Folic acid	Rare		hsa-mir-892c, hsa-mir-890, hsa-mir-888, hsa-mir-892a, hsa-mir-892b, hsa-mir-891b, hsa-mir-891a, hsa-mir-513c, hsa-mir-513b, hsa-mir-513a-1, hsa-mir-513a-2, hsa-mir-506, hsa-mir-507, hsa-mir-508, hsa-mir-514b, hsa-mir-509-2, hsa-mir-509-3, hsa-mir-509-1, hsa-mir-510, hsa-mir-514a-1, hsa-mir-514a-2, hsa-mir-514a-3	[150]
FRAXB	X	6,000,001	9,500,000	Xp22.31	Aphidicolin	Common		hsa-mir-4770, hsa-mir-4767, hsa-mir-651	[119]
FRAXC	X	98,300,001	102,600,000	Xq22.1	Aphidicolin	Common			[119]
FRAXD	X	140,300,001	142,100,000	Xq27.2	Aphidicolin	Common			[151]
FRAXE	X	147,100,001	155,270,560	Xq28	Folic acid	Rare	IKBKG	hsa-mir-2114, hsa-mir-4330, hsa-mir-224, hsa-mir-452, hsa-mir-105-1, hsa-mir-767, hsa-mir-105-2, hsa-mir-3202-1, hsa-mir-3202-2, hsa-mir-718, hsa-mir-6858, hsa-mir-664b, hsa-mir-1184-1, hsa-mir-1184-2, hsa-mir-1184-3	[152]
FRAXF	X	147,100,001	155,270,560	Xq28	Folic acid	Rare	IKBKG	hsa-mir-2114, hsa-mir-4330, hsa-mir-224, hsa-mir-452, hsa-mir-105-1, hsa-mir-767, hsa-mir-105-2, hsa-mir-3202-1, hsa-mir-3202-2, hsa-mir-718, hsa-mir-6858, hsa-mir-664b, hsa-mir-1184-1, hsa-mir-1184-2, hsa-mir-1184-3	[153]

A total of 125 CFSs were collected from GeneCards V3 [87] and from manual bibliographic search. Data for 1,871 human miRs were downloaded from miRBase 20 [53]. Genomic data of exon genes were downloaded from Ensembl Biomart [88]. A list of 327 genes that participate in Pathways in cancer (KEGG PATHWAY: hsa05200) was retrieved from KEGG [89]. PHP scripts parsed the data to populate a MySQL (http://www.mysql.com) relational database. Other scripts queried the database to produce a list of CFSs with their associated cancer-related genes and microRs. The main results found here are: (i) the total length of the cytogenetic bands of the 125 CFSs is 835.22 Mbps (27 % of the entire haploid genome), while 110 cancer-related genes (33.6 % of all cancer-related genes) and 686 miRs (36.7 % of all miRs) are located within these CFSs, (ii) the total length of the 26 CFSs with precise genomic boundaries is 79.3 Mbps (2.6 % of the entire haploid genome), and (iii) nine cancer-related genes (2.8 % of all cancer-related genes) and 44 miRs (2.4 % of all miRNAs) are located within these molecularly defined CFSs (Table 2). Based on the above, the density of cancer-related genes and miRs in cytogenetically defined CFS compared to the rest of the genome is 37.2 and 56.7 % higher, respectively

### Fragile sites and microRNA genes

According to an early study, miRs are particularly frequent in CFSs [6]. Out of the 186 miRs known at the time, 35 were found in, or very close (<3 Mb) to, CFSs, occurring at a density (number of miRs per length) that was estimated to be approximately 9 % higher than in non-FSs. A newer analysis has found approximately 33.8 % of 715 miRs within CFSs, corresponding to a relative 50 % (26–85 %) higher density in regard to non-FSs, but the relation seems to vary between chromosomes [7] with some, like chromosome 16 and 19, having many more fragile than non-fragile miRAs, and others, like chromosome 14, having a lower incidence of miRNAs in fragile regions. Given that the number of known miRs has more than doubled since then, we have repeated this analysis with the recent version of miRBase (v20, [53]) and have found 686 miRs out of 1,871 (36.7 %) within cytogenetically defined fragile sites (Table 2) (corresponding incidence in molecularly mapped CFSs is shown in Table 3). Thus, the relative density of miR in cytogenetically defined CFSs is 57 % higher than in the rest of the genome (Fig. 2a; Table 2). Specific pertinent examples include tumor suppressors like hsa-mir-34a in FRA1A and oncomirs like hsa-mir-21 in FRA17B. In addition, more than 50 % of microRNAs seem to be clustered in relatively short regions, up to 50 kb, often containing multiple miR isoforms belonging to the same family [54]. When mapped, approximately 28 % of miR clusters overlap with known FSs. Rearrangements within these regions can disrupt multiple miRs in a single hit and produce complex phenotypic changes.

**Table 3:** A list of molecularly delimited common fragile sites (CFSs) obtained from a manual search of the literature

FRA	Chr	Start	End	BAC/STS from	BAC/STS to	Type	Frequency	Cancer genes	miRNAs	References
FRA1E	1	97,749,961	98,119,925	RP4-538N4	RP11-128G13	Aphidicolin	Common			[92]
FRA1H	1	216,110,179	226,742,379	RP11-22M7	RP11-118H4	5AZA	Common	TGFB2	hsa-mir-215, hsa-mir-194-1, hsa-mir-664a, hsa-mir-320b-2, hsa-mir-4742, hsa-mir-6741	[154]
FRA2Ccen	2	18,524,760	19,272,430	RP11-720L11	RP11-78J22	Aphidicolin	Common			[97]
FRA2Ctel	2	14,934,889	15,681,539	RP11-526G2	RP11-32P22	Aphidicolin	Common			[97]
FRA2G	2	169,498,510	170,313,244	RP11-285F23	RP11-724O16	Aphidicolin	Common			[155]
FRA2H	2	186,716,111	187,251,132	RP11-561J1	RP11-639N24	Aphidicolin	Common			[156]
FRA3B	3	59,623,632	63,846,635	RP11-70P20	RP11-50F24	Aphidicolin	Common			[157]
FRA4F	4	90,208,191	97,312,924	RP11-549C16	RP11-145G20	Aphidicolin	Common			[109]
FRA6E	6	160,275,245	166,079,958	D6S1581	D6S1719	Aphidicolin	Common			[158]
FRA6F	6	111,577,970	112,568,919	RP5-1112D6	RP1-142L7	Aphidicolin	Common			[159]
FRA6H	6	27,804,038	37,156,663	RP1-193B12	RP11-588I14	Aphidicolin	Common	RXRB, PPARD, CDKN1A	hsa-mir-877, hsa-mir-4640, hsa-mir-6891, hsa-mir-6832, hsa-mir-4646, hsa-mir-1236, hsa-mir-6721, hsa-mir-6833, hsa-mir-3135b, hsa-mir-219a-1, hsa-mir-6873, hsa-mir-6834, hsa-mir-5004, hsa-mir-3934, hsa-mir-7159, hsa-mir-1275, hsa-mir-6835, hsa-mir-7111, hsa-mir-5690, hsa-mir-3925	[115]
FRA7B	7	1	12,392,296	Telomere	RP11-507C1	Aphidicolin	Common	PDGFA, RAC1	hsa-mir-339, hsa-mir-4655, hsa-mir-6836, hsa-mir-4648, hsa-mir-4656, hsa-mir-589, hsa-mir-6874, hsa-mir-3683	[116]
FRA7E	7	80,508,751	84,935,939	D7S1934	SHGC-104456	Aphidicolin	Common	HGF		[117]
FRA7G	7	115,894,865	116,072,877	D7S486	D7S522	Aphidicolin	Common			[160]
FRA7H	7	130,413,791	130,857,950	D7S786	D7S649	Aphidicolin	Common		hsa-mir-29a, hsa-mir-29b-1	[161]
FRA7I	7	144,671,106	146,121,417	SHGC-153624	sWSS2627	Aphidicolin	Common			[120]
FRA7K	7	110,657,856	111,031,412	WI-5281	SHGC-78648	Aphidicolin	Common			[121]
FRA8C	8	124,285,269	128,421,245	RP11-468O2	RP11-382A18	Aphidicolin	Common		hsa-mir-548aa-1, hsa-mir-548d-1, hsa-mir-6844, hsa-mir-4662b, hsa-mir-4662a	[162]
FRA9G	9	17,135,038	17,503,917	Within c9orf39/CNTLN		Aphidicolin	Common			[125]
FRA10F	10	125,391,503	128,256,865	RP11-391M7	RP11-179O22	Aphidicolin	Common	CTBP2	hsa-mir-4296, hsa-mir-4484	[130]
FRA11E	11	32,086,458	34,028,916	RP1-17K7	RP13-786C16	Aphidicolin	Common			[134]
FRA11G	11	113,688,799	118,157,704	RP11-667M19	RP11-832A4	Aphidicolin	Common	ZBTB16		[133]
FRA13A	13	35,546,088	36,184,897	RP11-307O13	RP11-270C18	Aphidicolin	Common			[139]
FRA13E	13	73,285,774	76,386,498	RP11-342J4	RP11-29G8	Aphidicolin	Common			[115]
FRA16D	16	78,420,359	78,731,553	AH009490.2	FRA16D seq	Aphidicolin	Common			[163]
FRAXB	X	6,595,111	7,548,235	DXS1130	DXS237	Aphidicolin	Common		hsa-mir-4767	[164]

The list of molecularly mapped CFSs was compiled by performing a systematic search of the literature for each one of the known cytogenetically mapped fragile sites (*n* = 125). Whenever the placement of STS markers or BACs on GRC37/hg19 was unknown, it was verified by megaBLAST (default parameters) of the complete sequence against the human chromosome sequences. Only matches with greater than 90 % coverage were considered for CFS placement. Twenty-six (26) CFSs with a precise mapping were identified (see Table 4), whose coordinates were then mapped to the reference genome version GRC37. Whenever the precise location of BACs was unknown, it was verified by alignment with NCBI Megablast (default settings). The annotation of the reference human genome was obtained from UCSC (URL: hgdownload.cse.ucsc.edu/goldenPath/hg19/) in December 2013. The utility “bigWigSummary” was used to extract mean scores for molecularly mapped fragile regions and randomly selected non-fragile control regions. The BAC/STS limits have been extracted from the cited publication and coordinates mapped on GRC37. Note that FRA2C has been divided into two separate, molecularly defined hotspots

### Fragile sites and regions with regulatory potential

Other DNA elements, such as regions with regulatory potential, may also be contained or overlap with CFSs. As an example, CTCF binding sites from ENCODE ChIP-seq are distributed throughout the genome. CTCF is a critical “weaver” of chromatin structure and function, and can provide an anchor point for nucleosome positioning [55]. Indirectly, CTCF can influence the accessibility of chromatin and plays diverse roles in chromatin insulation, gene regulation, imprinting, intra/interchromosomal interactions, nuclear compartmentalization, and alternative splicing [56, 57]. In some cases, distal fragments bound by CTCF have been found to mediate long-range interactions by loop formation and could modulate transcription at distant sites [58]. We examined the percentage of all potential CTCF binding sites within molecularly mapped CFSs and found that this ranges between 2.76 and 3.20 % in different cell lines (Table 4). Relative to the total length of fragile segments, this corresponds to an 18 % (10–25 %)-fold increase in the number of potential CTCF binding sites (Fig. 2a). Although it is impossible to know whether CTCF binding at these sites exerts a meaningful effect, its presence seems in accordance with the observation that many CFSs are gene-rich. Even more, CFSs rearrangements could influence gene expression further away on the same chromosome.

**Table 4:** Data from ENCODE [10] with respect to molecularly mapped common fragile sites (CFSs) from Table 3

Name	CTCF bs (count)	CTCF bs (%)	CTCF bs/kbp	H3K27ac	99-way cons. score
FRA1E	97	0.002	0.261	1.32	0.133
FRA1H	17,338	0.372	1.631	3.07	–
FRA2Ctel	700	0.015	0.938	2.53	0.071
FRA2Ccen	1,108	0.024	1.486	1.56	0.111
FRA2G	1,501	0.032	1.698	4.22	0.114
FRA2H	132	0.003	0.255	1.44	0.103
FRA3B	4,156	0.089	0.982	1.57	0.105
FRA4F	4,765	0.102	0.671	1.63	0.085
FRA6H	33,735	0.724	3.607	8.14	0.12
FRA6F	2,104	0.045	2.123	2.93	0.126
FRA6E	6,274	0.135	1.081	2.47	0.062
FRA7B	23,331	0.5	1.907	3.13	–
FRA7E	4,253	0.091	0.961	1.6	0.082
FRA7K	265	0.006	0.709	2.56	0.097
FRA7G	265	0.006	1.489	2.46	0.098
FRA7H	1,458	0.031	3.283	4.38	0.089
FRA7I	435	0.009	0.3	1.98	0.065
FRA8C	8,599	0.184	2.079	3.26	0.082
FRA9G	168	0.004	0.455	1.2	0.142
FRA10F	5,878	0.126	2.051	2.57	0.071
FRA11E	4,494	0.096	2.314	4.05	0.123
FRA11G	9,545	0.205	2.136	2.57	0.126
FRA13A	193	0.004	0.302	1.23	0.108
FRA13E	2,950	0.063	0.951	1.78	0.104
FRA16D	378	0.008	1.215	1.87	0.118
FRAXB	718	0.015	0.753	1.54	0.056

CTCF binding sites, studied in 89 cell lines, are shown respectively as absolute counts, with respect to the total number of CTCF binding sites in the whole genome and as a frequency per kb. Average H3k27 acetylation scores from ChIP-seq analysis of the K562 cell line have been calculated for each fragile site. Average conservation score between human and 99 vertebrates was obtained from the UCSC browser (http://genome.ucsc.edu/cgi-bin/hgTrackUi?db=hg19&g=cons100way). We compiled a list of molecularly mapped CFSs by performing a systematic search of the literature for each one of the known cytogenetically mapped CFSs (*n* = 125). Twenty-six CFSs with a precise mapping were identified (see Table 3), and their coordinates were then mapped to the reference genome version GRC37. Whenever the precise location of BACs was unknown, it was verified by alignment with NCBI Megablast (default settings). The annotation of the reference human genome was obtained from UCSC (URL: hgdownload.cse.ucsc.edu/goldenPath/hg19/) in December 2013. The utility “bigWigSummary” was used to extract mean scores for molecularly mapped fragile regions and randomly selected non-fragile control regions

### Fragile sites and histone modifications

Epigenetic modifications, such as histone methylation and acetylation, can also take place within FS. It appears that H3K9/14 hypoacetylation is a global feature of CFSs in a lymphoblastoid cell line [9] and could impede replication progression. Several enzymes modify key histone residues with relatively high specificity and regulate, indirectly, transcription, repair, and replication. Indeed, histone modifications vary significantly between cell types and are well correlated with transcription levels in the ENCODE data (Figure 2 in [10]), especially for H3K79me2, H3K9ac, H3K4me3, and H3K27ac. Histone 3 lysine 27 acetylation (H3K27ac) co-localizes with active enhancers [59] and regions with open chromatin structure and could play a role in protecting against replication–transcription collisions and R-loop formation. Intriguingly, H3K27ac varies between cell types and CFSs (Fig. 2b, c). The average ChIP-seq acetylation signal and the number of signal peaks (data not shown) within cytogenetic CFSs are slightly lower than the corresponding chromosome mean in K562 cells, but higher in HUVEC cells and equivocal for other cell types. Such variability may explain the plasticity of CFSs and their differential expression between individuals, cell types, and culture conditions.

Histone 3 lysine 4 trimethylation is also associated with active promoters [60] and correlates well with transcription in the ENCODE data [10]. It appears that H3K4me3 may also contribute to the DNA damage response and repair of DSBs in yeast cells [61], mediating cellular responses to genotoxic stresses, and interacting with the human tumor suppressor ING1, which is required for DNA repair and apoptotic activities [62]. A recent study of ERFSs [9] has shown that replication stall, as identified by anti-replication protein A ChIP, preferentially co-localizes with H3K4me3 (see supplemental figure 1 in [16]). Although cytogenetically defined CFSs as a whole do not show a large deviation from the mean, some sites in particular, like FRA3B and FRA16D, seem to be on average poor in H3K4me3 while others, like FRA2E, FRA3C, and FRA7D seem to be on average rich in H3K4me3 (Fig. 2c). When the cell lines employed were grouped according to their origin (cancerous versus embryonic versus normal epithelial versus normal mesenchymal), a pattern regarding the density of the H3K27ac and H3K4me3 within CFSs relative to non-fragile sites could be discerned (Fig. 2b). Cancer and embryonic cells (K562, GM12878, H1-hESC) displayed lower signals of H3K27ac and H3K4me3 relative to the non-fragile regions, whereas a significantly different distribution was noticed in the other cell line groups (*p* < 0.001, ANOVA). Despite the small number of cell lines examined, a possible functional link between histone modifications and the other elements (genes, non-coding RNAs, regulatory sequences) positioned within the CFS cannot be excluded (Fig. 1). This potential interplay may be even more complex during carcinogenesis. Oncogenes may distress this functional cross-talk by altering the epigenome of a particular region. As an example, oncogenic Cdc6 was shown to act as “molecular switch” at certain tumor-suppressor loci by regulating CTCF binding. The latter led to suppression of the genes encoded and simultaneous firing of adjacent dormant origins. If such a scenario takes place within CFS that are rich in CTCF sites (Fig. 2a), and depending on the cellular context, the density and timing of firing origins can be altered affecting replication dynamics [56, 57].

Our current understanding of CFSs is traditionally based on a static mapping, often cytogenetic and imprecise, which cannot fully capture the interaction of non-coding DNA, regulatory elements, and histone modifications with vulnerability to RS. Even though the current CFS mapping successfully predicts response to extrinsic stress and OIRS, a more accurate model of fragility will eventually have to integrate experimental data at the nucleotide resolution with other non-coding elements. This is an intriguing area for further study.

## Fragile sites in carcinogenesis

Extending the concept of CFSs in carcinogenesis has been a subject of active research since the discovery of an association between cancer breakpoints and FSs [63]. Overall, it appears that CFSs are generally sensitive to innate RS occurring naturally in various tumors and cell lines [64]. Multiple clusters of homozygous deletions, usually small, have been detected over known CFSs in an exhaustive survey of cancer genomes but their expression profile is variable [65]. For example, FRA2F, FRA3B, FRA4F, FRA5H, and FRA16D were most affected while others, like FRA2B and FRA4B, were least affected. Recurrent alterations have been identified in FRA3B and FRA16D in several cancer types, leading to further investigation of the *FHIT* and *WWOX* genes, respectively, in mouse models (see K. Huebner and R. Aqeilan chapters in this issue). Fragile sites FRA10C and FRA10G may be involved in the formation of the oncogenic *RET/PTC* rearrangement in papillary thyroid carcinoma [15]. Specifically, in *RET/PTC1*, the FRA10G-localized *RET* is rearranged with the FRA10C-localized tumor suppressor gene *CCDC6*, while in RET/PTC3 it is rearranged with NCO4 that is located in FRA10G. In a similar manner, the *MYC* oncogene is flanked by CFSs FRA8C and FRA8D, that may facilitate adjacent integration of HPV18 [66] or *MYC* amplification [67]. Viral DNA integration in the genome of a host cell can lead to cancer development and CFSs provide preferential hotspots for this [68]. Particularly, HPV16 E6 and E7 oncogenic products have been shown to induce replication stress and DSBs in the host cell. This occurs preferentially at CFSs allowing viral genome integration at these sites [68].

Despite the abundance of CFS breaks in cancer, it would be inappropriate to assume that alterations of genes residing within CFSs always confer a clonal advantage in cancer development [65] without evidence of selection or at least convincing causative models. Clearly, breakage probability (passenger alterations) as a consequence of fragility and clonal selection (driver alterations) in cancer development are two separate phenomena that should not be confused.

Nevertheless, the impact of CFS instability in cancer should not be easily dismissed or oversimplified. CFSs breakpoints have been detected in preneoplastic lesions in human and mouse models [4, 19] well before the emergence of the malignant phenotype. Briefly, exposure of xenografted normal human skin to growth factors preferentially induces CFS instability. Similarly, hyperplastic mouse urothelium from *HRAS* transgenic mice showed numerous copy number alterations in fragile areas. CFS instability is an early manifestation and can be attributed to experimentally controlled, oncogene-induced stress in these studies, in a way that more closely resembles carcinogenesis than APH-induced stress. Therefore, it could be argued that CFS alterations are frequent in cancer, as described above [65], not just because of a higher breakage probability but also because of an earlier involvement, even before the complete deregulation of the cellular machinery.

Furthermore, any double-strand break can have dire consequences, such as the initiation of a breakage-fusion-bridge cycle, especially when subtelomeric and peri-centromeric CFSs are disrupted simultaneously [69]. Through this mechanism, CFS breaks can amplify oncogenes, delete tumor suppressors or, most importantly, initiate persistent chromosomal instability. Massive accumulation of localized chromosomal rearrangements in a single time-point, termed chromothripsis (literally: shattering of the chromosome) and chromoanasynthesis, has recently been identified in several cancer types [70]. Indirect evidence suggests that CFSs may have an important role in this process [71] by stalling the RF, favoring RF collapse and, in extreme cases, chromosome pulverization leading to clustering of chromosomal breaks [13]. Indeed, chromosome fragmentation distal to the CFS has been observed under the microscope in some cases [71] and could be a triggering factor for chromothripsis. On the other hand, multi-step, recurrent CFS alterations could be difficult to discriminate from single-step rearrangements, rendering the identification of chromothripsis even more difficult. In that scenario, CFS stability and localization is an important parameter in the bioinformatic algorithms that are applied to define and model such cancer rearrangements. In addition, CFSs can contribute to the clustered shuttering of the chromosomes also during the process of premature chromatin condensation (PCC) [72, 73] in which interphase chromosomes or late replicating chromosome zones like CFS or extranuclear bodies micronuclei are ‘induced’ to condensation by various mitotic factors [74]. This reveals more possibilities for CFSs to act as contributors to GI through chromosome breakage. An interesting scenario suggested that the G2–M mammalian checkpoint can fail to delay mitotic onset as it may not be sensitive enough to detect a few remaining long-replicating forks, thus allowing chromatin condensation of late replicating CFS regions, resulting in multiple DNA breaks [26, 44].

## CFSs as “functional” units: a new perception

**Fig. 3:**
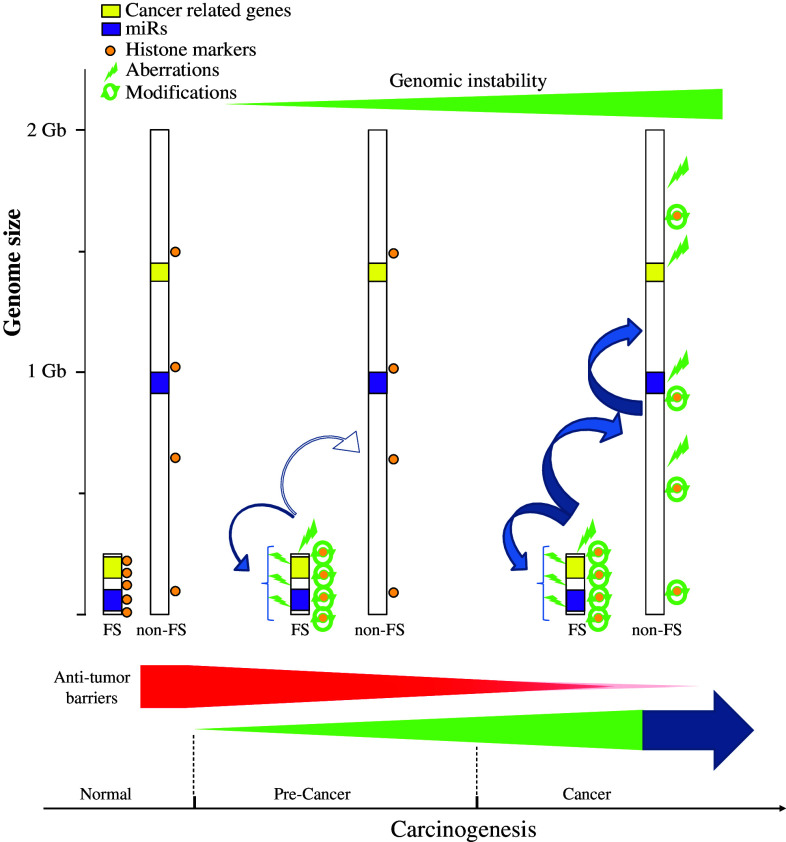
Model proposing that CFS apart from contributing to GI exert wider biological effects during cancer development. CFSs are preferentially affected from the earliest precancerous lesions, in response to OIRS, conferring to GI. A wide spectrum of coding and non-coding elements are present within CFSs. Cancer-related genes and miRs may be affected from such early precancerous stages, therefore possibly exerting a strong pressure for malignant progression. This pressure is also reinforced by alterations and imbalances in the binding elements and histone patterns, respectively, in the CFSs. Furthermore, collectively, all of these alterations may further affect in an “avalanche” mode not only the stability of the CFSs, but overall of the genome. As the anti-tumor barriers are gradually overwhelmed, this avalanche effect may function in a positive feedback mode to promote cancer

Common fragile sites have long been considered vulnerable breakage sites in the genome in response to RS from extrinsic factors. Their fragility has also been associated with GI in cancer development. As we have previously shown, CFSs are preferentially affected from the earliest precancerous lesions, in response to OIRS [2, 4]. In the current work, we first performed a review on the heterogeneity and fragility mechanisms affecting these sites. Next, by applying bioinformatic tools and exploiting available information in various databases, like the KEGG, miRbase, and ENCODE, we show a prevalence of various cancer-related genes, miRs, binding elements, and histone modifications in CFSs (Figs. 1, 3). The presence of such a wide spectrum of coding and non-coding elements changes the view on CFSs content and their nature itself. Given that CFSs are altered from the earliest stages in cancer, their impact on cancer development may be more profound than simply participating in the emergence of GI. On one hand, cancer-related genes and miRs may be affected from such early precancerous stages, therefore possibly exerting a strong pressure for malignant progression (Fig. 3). On the other hand, this pressure is also reinforced by alterations and imbalances in the binding elements and histone patterns, respectively, in the CFSs. Furthermore, collectively, all these alterations may further affect in an “avalanche” mode not only the stability of the CFSs, but overall of the genome (Figs. 1, 3). Therefore, as the anti-tumor barriers are gradually overwhelmed, this avalanche effect may function in a positive feedback mode to promote cancer. An important question that emerges is why CFSs are not selected for elimination from the genome, but are rather conserved features in mammals? A tempting but speculative answer is that by locating a set of important coding and non-coding elements in regions that replicate late and/or with delay and thus are prone to instability, they may function as alarm sensors scattered throughout the genome in various chromosomes, to signify detrimental effects from the RS on the cell. As long as the mammalian checkpoints and repair mechanisms are not compromised, cells can monitor and protect their genome and functional integrity through such a dynamic interaction. Nevertheless, this imposes the risk that if the checkpoints and the anti-tumor barriers gradually fail, tumor promotion ensues (Fig. 3). As we were able to examine only a small subset of binding elements and histone modifications from the ENCODE and the miRbase is constantly expanding, in the future more in-depth studies are required to obtain a comprehensive picture of CFSs and on their role in cancer. Overall, CFSs may not be merely structural domains vulnerable only to breakage but highly organized “functional” units that may have deeper biological consequences for the cell when affected.

## Footnotes

1
http://www.genome.jp/kegg/pathway/hsa/hsa05200.html.
